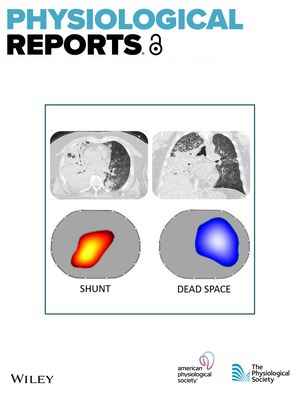# Cover Image

**DOI:** 10.14814/phy2.70345

**Published:** 2025-07-15

**Authors:** Roberta Garberi, Claudio Ripa, Gianmarco Carenini, Luca Bastia, Marco Giani, Giuseppe Foti, Emanuele Rezoagli

## Abstract

The cover image is based on the article Personalized ventilation guided by electrical impedance tomography with increased PEEP improves ventilation‐perfusion matching in asymmetrical airway closure and contralateral pulmonary embolism during veno‐venous extracorporeal membrane oxygenation: A case report by Emanuele Rezoagli et al., https://doi.org/10.14814/phy2.70280